# Physiological Chemistry

**Published:** 1891-09

**Authors:** John A. Miller

**Affiliations:** Ph. D. (Berlin)


### Progress in Medical Science.

### PHYSIOLOGICAL CHEMISTRY.

CONDUCTED BY

JOHN A. MILLER, Ph. D. (BERLIN).

### THE GERMICIDAL ACTION OF EGG ALBUMIN.

A. WURTZ (*Compt. Rend. Soc. Biolog.*, 42, 20 ; *Ber. Deut. Chem. Ges.*, 24, 465,) found that by the digestion with the uncooked white of egg at 38° a small number of bacillus anthrax, cholera-spirillum, Ebert's bacillus, bacillus pyrogenes aureus, and of bacillus subtilis were killed. This property of the albumin prevents the invasion of eggs by bacteria. Coagulated egg albumin has no germicidal properties.

### INFLUENCE OF ALKALIES ON THE FORMATION OF GLYCOGEN IN THE LIVER.

E. DEFOURT (*Compt. Rend. Soc. Biolog.*, 42, 146 ; *Ber. Deut. Chem. Ges.*, 24, 466,) finds that after the administration of sodium bicarbonate to dogs and guinea-pigs, the total contents of sugar in the liver was in excess of the normal.

### OXYGEN IN THE BLOOD OF ANIMALS AT GREAT ALTITUDES.

VIAULT (*Compt. Rend.*, 112, 295 ; *Jour. Chem. Soc.*, 60, 753,) finds that at great altitudes the proportion of oxygen in the blood of animals is practically the same as at low levels. It would seem that the greater rarefaction of the oxygen is compensated by the greater degree of subdivision of the red corpuscles and their consequent greater absorptive activity.

### EFFECT OF MEDICINES, AND ESPECIALLY OF VALERIAN EXTRACT, ON THE DESTRUCTION OF DEXTROSE IN THE BLOOD.

L. BUTTE (*Compt. Rend.*, 112, 347 ; *Jour. Chem. Soc.*, 60, 754). Sodium bicarbonate and morphine retards the destruction of dextrose in the blood, but curarine accelerates it. Valerian extract retards the destruction very considerably when added to freshly drawn blood, and also when injected into the left femoral vein of a dog.

---

### ISOLATION OF THE GLYCOLITIC FERMENT OF THE BLOOD.

R. Lepine and Barral (*Compt. Rend.*, 112, 411; *Jour. Chem. Soc.*, 60, 755). Fresh dog's blood was defibrinated and placed in a powerful centrifugal machine; the serum thus separated was found to have very little glycolitic power. The separated corpuscles were treated with a volume of salt water equal to the original serum, and the process of separation repeated; the salt solution contained less albumin than the serum, but its glycolitic power was much greater. A second treatment with salt water gave a liquid with very little albumin, but with still greater glycolitic power. It follows, therefore, that the destruction of glucose is not due, as Arnaud has supposed, to some action of living blood albumin; the results point, in fact, to the existence of a soluble ferment.

---

### PATHOLOGY OF PROTEIDS.

S. Martin (*Brit. Med. Jour.*, 1, 1891; *Jour. Chem. Soc.*, 60, 761). The changes in structure that occur in a diseased tissue or organ are associated with chemical changes, and it is to certain changes in the proteids, the most abundant constituent of most living structures, that attention was chiefly directed in the present research; although peptones and albumoses are found in the alimentary canal, their presence elsewhere in the body is pathological, as during normal absorption they are regenerated into the blood proteids. The action of microörganisms during putrefaction is, first, similar to that of pepsin, namely: it forms albumoses and peptones, but, subsequently, ptomaines may make their appearance. Putrefaction (using the word in the wide sense of the changes due to microbes) is, moreover, a frequent factor in pathological processes, and albumoses and peptones are poisonous substances. If these poisons are found in decomposing collections of cells, as in abscesses, empyema, phthisis, etc., they pass into the lymph and blood-streams, passing ultimately from the body by the urine, constituting what is generally termed peptonuria, but which should be more properly called albumosuria, the proteid which is present being deutero-albumose. Illustrative cases are cited which show the amount of albumose in the pus and in the urine before and after the evacuation of the abscess, the albumose in the urine varying with the amount of accumulated pus.

One of the most striking features of pus-formation which is common to it and albumose poisoning is fever. It is possible that other factors have also to be considered ; thus leucine, often found in pus, is a fever producer ; alkaloids, if they do exist, have yet to be isolated.

In ordinary pus, the agent which forms the albumoses is probably not an euzyme, but the *shapely lococcus pyogenes aureus ;* in tubercle, it may be the tubercle bacillus.

But the presence of albumoses in disease cannot always be ascribed to microörganisms, as in glycosuria and osteomalacia. In osteomalacia the diseased bones contain an albumose similar to that excreted in the urine, and, perhaps, due to the rapid breaking down of lowly organized cells. In the puerperal state, the origin of the albumose may be the degenerating cells of the hypertrophied uterus which is undergoing involution. The origin of albumoses in other cases, such as measles, is at present involved in obscurity.

---

### CULTIVATION PRODUCTS OF THE TUBERCLE BACILLUS.

E. M. Crookshank and E. F. Herronn (*Brit. Med. Jour.*, 1, 1891; *Jour. Chem. Soc.*, 60, 762). In cultivations of the tubercle bacillus in glycerol broth, albumoses, substances of the nature of albumose and peptone were identified, and also a substance which gives the reactions of an alkaloid. The latter was, however, obtained in too small a quantity for more than a qualitative analysis. Injected into animals, symptoms similar to those produced by Koch's tuberculin were obtained.

